# Crystal structures of (5*RS*)-(*Z*)-4-[5-(furan-2-yl)-3-phenyl-4,5-di­hydro-1*H*-pyrazol-1-yl]-4-oxobut-2-enoic acid and (5*RS*)-(*Z*)-4-[5-(furan-2-yl)-3-(thio­phen-2-yl)-4,5-di­hydro-1*H*-pyrazol-1-yl]-4-oxobut-2-enoic acid

**DOI:** 10.1107/S2056989016013992

**Published:** 2016-10-11

**Authors:** Kseniya K. Borisova, Flavien A. A. Toze, Nniyaz Z. Yagafarov, Fedor I. Zubkov, Pavel V. Dorovatovskii, Yan V. Zubavichus, Victor N. Khrustalev

**Affiliations:** a Organic Chemistry Department, Peoples’ Friendship University of Russia (RUDN University), 6 Miklukho-Maklay St., Moscow 117198, Russian Federation; b Department of Chemistry, Faculty of Sciences, University of Douala, PO Box 24157, Douala, Republic of , Cameroon; c National Research Centre "Kurchatov Institute", 1 Acad. Kurchatov Sq., Moscow 123182, Russian Federation; d Inorganic Chemistry Department, Peoples’ Friendship University of Russia (RUDN University), 6 Miklukho-Maklay St., Moscow 117198, Russian Federation; e X-Ray Structural Centre, A.N. Nesmeyanov Institute of Organoelement Compounds, Russian Academy of Sciences, 28 Vavilov St., B-334, Moscow 119991, Russian Federation

**Keywords:** crystal structure, intra­molecular Diels–Alder reaction, furan, thio­phene

## Abstract

The title compounds, C_17_H_14_N_2_O_4_ (I) and C_15_H_12_N_2_O_4_S (II), possess very similar mol­ecular geometries. In both mol­ecules, the central 1,3,5-tris­ubstituted di­hydro­pyrazole ring adopts an envelope conformation. The oxobutenoic acid fragment has an almost planar *Z* conformation [r.m.s. deviations of 0.049 and 0.022 Å, respectively, for (I) and (II)] which is determined by the both bond conjugation and the strong intra­molecular O—H⋯O hydrogen bond. The substituents in positions 1 and 3 of the di­hydro­pyrazole ring [oxobutenoic acid and phenyl in (I) and oxobutenoic acid and thienyl in (II)] are nearly coplanar with its basal plane [the corresponding dihedral angles are 6.14 (9) and 2.22 (11)° in (I) and 6.27 (12) and 3.91 (11)° in (II)]. The furyl ring plane is twisted relative to the basal plane of the di­hydro­pyrazole ring by 85.51 (8) and 88.30 (7)° in (I) and (II), respectively. In the crystal of (I), mol­ecules form zigzag hydrogen-bonded chains along [001] by C—H⋯O hydrogen bonds, which are further packed in stacks along [100]. Unlike (I), the crystal of (II) contains centrosymmetric hydrogen-bonded dimers formed by pairs of C—H⋯S hydrogen bonds, which are further linked by weak C—H⋯O hydrogen bonds into a three-dimensional framework.

## Abstract 2

Stereochemical peculiarities of (5*RS*)-(*Z*)-4-[5-(furan-2-yl)-3-phenyl-4,5-di­hydro-1*H*-pyrazol-1-yl]-4-oxobut-2-enoic acid and (5*RS*)-(*Z*)-4-[5-(furan-2-yl)-3-(thio­phen-2-yl)-4,5-di­hydro-1*H*-pyrazol-1-yl]-4-oxobut-2-enoic acid, studied by X-ray structural analysis, render impossible their transformation into 3b,6-ep­oxy­pyrazolo­[5,1-*a*]iso­indoles by a thermal intra­molecular Diels–Alder reaction of furan (the IMDAF reaction).

## Chemical context   

**Figure 1:**
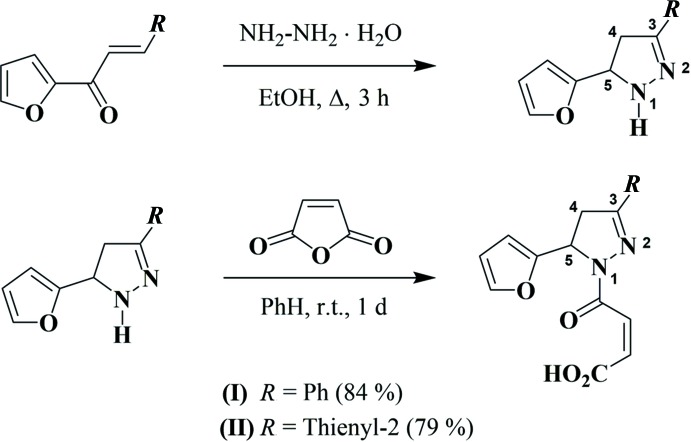
Synthesis of maleic amides (I) and (II) from (*E*)-1-(furan-2-yl)-3-aryl­prop-2-en-1-ones.

**Figure 2:**
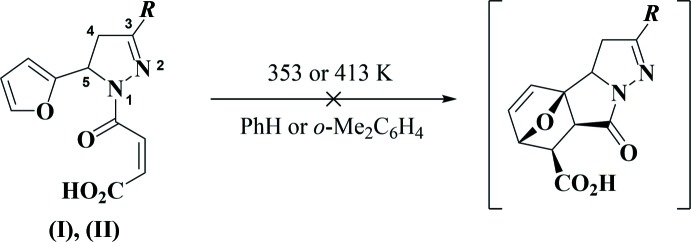
The purposed thermal IMDAF reaction of maleic amides (I) and (II).

3-(2-Fur­yl)pyrazolines and their *N*-acyl derivatives are well known to possess high and diverse biological activity, for example, topoisomerase I and II inhibitory and anti­proliferative activity (Ahmad *et al.*, 2016 ▸), 5α-reductase inhibi­tory activity (Banday *et al.*, 2014 ▸), anti­bacterial (Joshi *et al.*, 2016 ▸; Bhoot *et al.*, 2012 ▸), anti­tuberculous (Manna & Agrawal, 2010 ▸), anti-inflammatory (Shoman *et al.*, 2009 ▸), anti­fungal activity (Deng *et al.* 2012 ▸), and many others. Pyrazolines, fused with other heterocycles, are much less studied. Thus, the main goal of this work was the synthesis of maleic amides (I) and (II) from (*E*)-1-(furan-2-yl)-3-aryl­prop-2-en-1-ones (Fig. 1 ▸) with subsequent their transformation into 3b,6-ep­oxy­pyrazolo­[5,1-*a*]iso­indoles by a thermal intra­molecular Diels–Alder reaction of furan (the IMDAF reaction). However, we were unable to realize the final stage of the purposed synthesis – the thermal IMDAF reaction of maleic amides (I) and (II) (Fig. 2 ▸). Unexpectedly, these compounds remained unchanged at temperatures up to 413 K. In order to explain this fact by an understanding of their stereochemical features, an X-ray diffraction study of compounds (I) and (II) was undertaken.
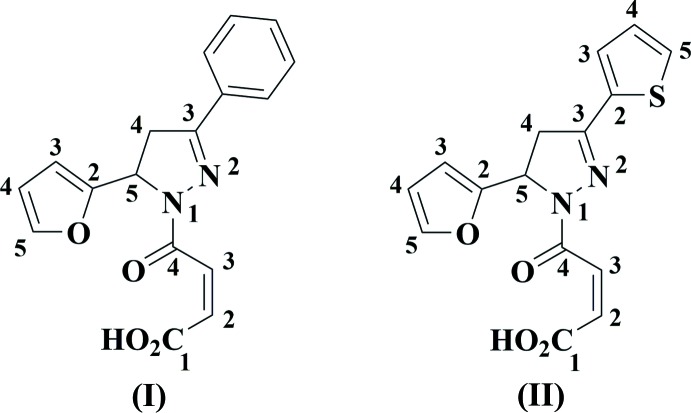


## Structural commentary   

**Figure 3:**
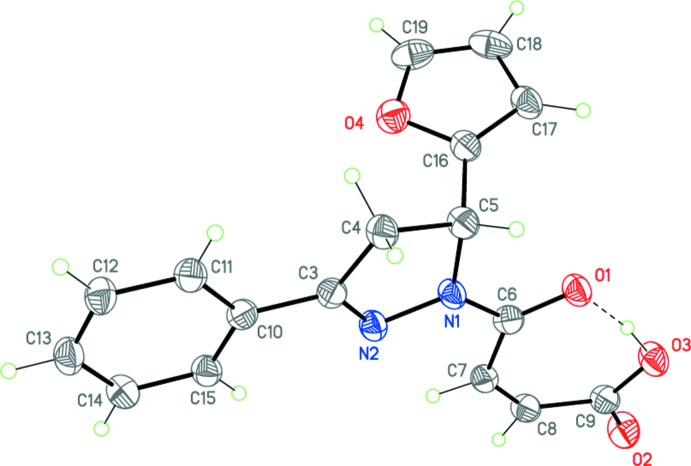
The mol­ecular structure of (I). Displacement ellipsoids are shown at the 50% probability level. H atoms are presented as small spheres of arbitrary radius. The dashed line indicates the intra­molecular O—H⋯O hydrogen bond.

Compounds (I), C_17_H_14_N_2_O_4_, and (II), C_15_H_12_N_2_O_4_S, possess very similar mol­ecular geometries (Figs. 3 ▸ and 4 ▸). In both mol­ecules, the central 1,3,5-tris­ubstituted di­hydro­pyrazole ring adopts an envelope conformation, with the C5 carbon atom deviating from the plane through the other atoms of the ring by 0.251 (3) and 0.178 (3) Å, respectively, in (I) and (II). The oxobutenoic acid fragment has an almost planar *Z* conformation [r.m.s. deviations of 0.049 and 0.022 Å, respectively, for (I) and (II)] which is determined by both the bond conjugation and the strong intra­molecular O3—H3⋯O1 hydrogen bond (Tables 1 ▸ and 2 ▸, Figs. 3 ▸ and 4 ▸). The substituents in positions 1 and 3 of the di­hydro­pyrazole ring [oxobutenoic acid and phenyl in (I) and oxobutenoic acid and thienyl in (II)] are practically coplanar with its basal plane [the corresponding dihedral angles are 6.14 (9) and 2.22 (11)° in (I) and 6.27 (12) and 3.91 (11)° in (II)]. Importantly, the furyl ring plane is twisted relative to the basal plane of the di­hydro­pyrazole ring by 85.51 (8) and 88.30 (7)° in (I) and (II), respectively. Apparently, it is such a perpendicular arrangement of the furyl and oxobutenoic acid fragments that inhibits the IMDAF reaction between them.

The nitro­gen atom N1 has a planar–trigonal geometry, the sum of the bond angles being 359.9° for (I) and 360.0° for (II). The bond lengths and angles in (I) and (II) are in good agreement with those observed in related structures (Suponitsky *et al.*, 2002 ▸; Guo, 2007 ▸; Vinutha *et al.*, 2013 ▸). The mol­ecules possess an asymmetric center at the C5 carbon atom. The crystals of (I) and (II) are racemic and consist of (5*RS*)-enanti­omeric pairs.

## Supra­molecular features   

Although the similarity of the mol­ecular geometries and intra­molecular inter­actions might lead to similar packing motifs, this is not found in the case for (I) and (II). The inter­molecular inter­actions, namely C—H⋯O and C—H⋯S hydrogen bonding, combined in a different way, give rise to different packing networks.

**Figure 5:**
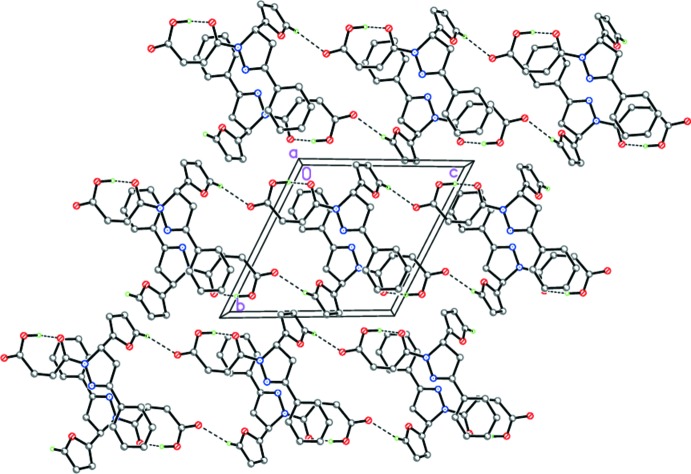
The crystal structure of (I) showing the hydrogen-bonded chains along [001]. Dashed lines indicate the intra­molecular O—H⋯O and inter­molecular C—H⋯O hydrogen bonds.

In the crystal of (I), mol­ecules form zigzag hydrogen-bonded chains along [001] by the C19—H19⋯O2(*x*, *y*, *z* − 1) hydrogen bonds (Table 1 ▸ and Fig. 5 ▸), which are further packed in stacks along [100] (Fig. 5 ▸).

**Figure 6:**
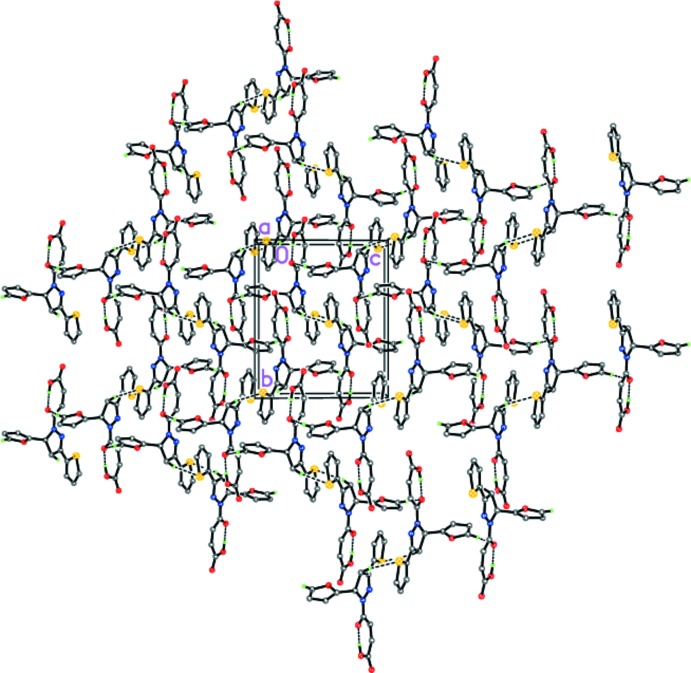
The crystal structure of (II) along the *a* axis. Dashed lines indicate the intra­molecular O—H⋯O and inter­molecular C—H⋯S and C—H⋯O hydrogen bonds.

However, unlike in (I), the crystal of (II) contains centrosymmetric hydrogen-bonded dimers formed by the two C4—H4*B*⋯S1(−*x* + 1, −*y*, −*z* + 2) hydrogen bonds (Table 2 ▸ and Fig. 6 ▸), which are further linked by weak C17—H17⋯O1(*x* − 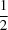, −*y* + 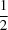, *z* − 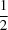) hydrogen bonds into a three-dimensional framework (Table 3 ▸ and Fig. 6 ▸).

## Synthesis and crystallization   

The initial 5-(furan-2-yl)-3-aryl-4,5-di­hydro-1*H*-pyrazoles were synthesized from (*E*)-1-(furan-2-yl)-3-aryl­prop-2-en-1-ones according to the procedure described previously (Grandberg *et al.* 1960 ▸; Kriven’ko *et al.* 2000 ▸; Cetin *et al.* 2003 ▸; Özdemir *et al.* 2007 ▸).

**General procedure**. A solution of the corresponding (*E*)-1-(furan-2-yl)-3-aryl­prop-2-en-1-one (0.025 mol) in alcohol (15 mL) was added to a solution of hydrazine hydrate (2.5 mL, 0.05 mol) in alcohol (15 mL). The mixture was heated at reflux for 3–5 h (TLC monitoring), then the solvent and the excess of hydrazine hydrate were removed under reduced pressure. The residue, viscous brown oil, was dissolved in benzene (15 mL) and acyl­ated (stirring at room temperature for 1 day) with a solution of maleic anhydride (2.45 g, 0.025 mol) in benzene (25 mL). The precipitated crystals were filtered off and recrystallized from an EtOH–DMF mixture to give the analytically pure maleic amides (I) and (II).

**(5** ***RS*** **)-(** ***Z*** **)-4-[5-(Furan-2-yl)-3-phenyl-4,5-di­hydro-1** ***H*** **-pyra­zol-1-yl]-4-oxobut-2-enoic acid (I)**. Colourless rhombic prisms. Yield is 4.88 g (63%). M.p. = 453.7–455.6 K with decomp. (EtOH–DMF). ^1^H NMR (DMSO, 600 MHz, 303 K): δ = 3.40 (*dd*, 1H, H4*A*, *J*_4,4_ = 17.7, *J*_4A,5_ = 5.0), 3.76 (*dd*, 1H, H4*B*, *J*_4,4_ = 17.7, *J*_4B,5_ = 11.8), 5.70 (*dd*, 1H, H5, *J*_5,4A_ = 5.0, *J*_4B,5_ = 11.8), 6.29 (*d*, 1H, —CH=CH—CO_2_H, *J* = 12.1), 6.39–6.41 [*m*, 2H, H3 and H4 (fur­yl)], 6.91 (*d*, 1H, —CH=CH—CO_2_H, *J* = 12.1), 7.44–7.49 [*m*, 3H, H3, H4 and H5 (Ph)], 7.57 [*m*, 1H, H5 (fur­yl)], 7.77–7.79 [*m*, 2H, H2 and H6 (Ph)], 12.29 (*br s*, 1H, CO_2_H). ^13^C NMR (DMSO-*d_6_*, 150.9 MHz, 303 K): δ = 38.8 (C4), 54.0 (C5), 107.9 and 111.0 [C3 and C4 (fur­yl)], 127.3 [2C, C3 and C5 (Ph)], 129.4 [2C, C2 and C6 (Ph)], 130.1, 131.0, 131.17 [C1 (Ph)], 131.20, 143.0 [C5 (Fur­yl)], 152.6, 156.2, 163.0, 167.4.

**(5** ***RS*** **)-(** ***Z*** **)-4-[5-(Furan-2-yl)-3-(thio­phen-2-yl)-4,5-di­hydro-1** ***H*** **-pyrazol-1-yl]-4-oxobut-2-enoic acid (II)**. Light-yellow rhombic prisms. Yield is 4.03 g (51%). M.p. = 449.8–450.9 K with decomp. (EtOH–DMF). ^1^H NMR (DMSO, 600 MHz, 301 K): δ = 3.41 (*dd*, 1H, H4*A*, *J*_4,4_ = 17.5, *J*_4A,5_ = 4.4), 3.76 (*dd*, 1H, H4*B*, *J*_4,4_ = 17.5, *J*_4B,5_ = 11.8), 5.69 (*dd*, 1H, H5, *J*_5,4A_ = 4.4, *J*_4B,5_ = 11.8), 6.30 (*d*, 1H, —CH=CH—CO_2_H, *J* = 12.1), 6.39–6.41 [*m*, 2H, H3 and H4 (fur­yl)], 6.81 (*d*, 1H, —CH=CH—CO_2_H, *J* = 12.1), 7.16 [*dd*, 1H, H4 (thien­yl), *J*_3,4_ = 3.5, *J*_4,5_ = 4.9], 7.52 [*dd*, 1H, H3 (thien­yl), *J*_3,4_ = 3.5, *J*_3,5_ = 1.3], 7.57 [*m*, 1H, H5 (fur­yl), 7.75 [*dd*, 1H, H5 (thien­yl), *J*_3,5_ = 1.3, *J*_4,5_ = 4.9], 12.8 (*br s*, 1H, CO_2_H). ^13^C NMR (DMSO, 150.9 MHz, 301 K): δ = 39.5 (C4), 54.1 (C5), 108.0, 111.1, 128.7, 130.3, 130.6, 130.8, 131.4, 134.2 [C1 (Ph)], 143.1 [C5 (fur­yl)], 152.1, 152.4, 162.7, 167.3.

## Refinement   

Crystal data, data collection and structure refinement details are summarized in Table 3 ▸. X-ray diffraction studies were carried out on the ‘Belok’ beamline (λ = 0.96990 Å) of the National Research Center "Kurchatov Institute" (Moscow, Russian Federation) using a MAR CCD detector. For each compound a total of 360 images were collected using an oscillation range of 1.0° (*φ* scan mode, two different crystal orientations) and corrected for absorption using the *SCALA* program (Evans, 2006 ▸). The data were indexed, integrated and scaled using the utility *i*MOSFLM in *CCP4* (Battye *et al.*, 2011 ▸).

The hydrogen atoms of the hydroxyl groups were localized in difference-Fourier maps and refined in an isotropic approximation with fixed displacement parameters [*U*_iso_(H) = 1.5*U*_eq_(O)]. The other hydrogen atoms were placed in calculated positions with C—H = 0.95–1.00 Å and refined using a riding model with fixed isotropic displacement parameters [*U*_iso_(H) = 1.2*U*_eq_(C)].

The insufficient data completeness of 96.7% in the case of (I) is determined by the low (triclinic) crystal symmetry. It is very difficult to get good data completeness at this symmetry using the *φ* scan mode only (‘Belok’ beamline limitation), even though we have run two different crystal orientations.

## Supplementary Material

Crystal structure: contains datablock(s) global, I, II. DOI: 10.1107/S2056989016013992/xu5892sup1.cif

Structure factors: contains datablock(s) I. DOI: 10.1107/S2056989016013992/xu5892Isup2.hkl

Structure factors: contains datablock(s) II. DOI: 10.1107/S2056989016013992/xu5892IIsup3.hkl

Supporting information file. DOI: 10.1107/S2056989016013992/xu5892Isup4.cml

Supporting information file. DOI: 10.1107/S2056989016013992/xu5892IIsup5.cml

CCDC references: 1502198, 1502197

Additional supporting information:  crystallographic information; 3D view; checkCIF report

## Figures and Tables

**Figure 4:**
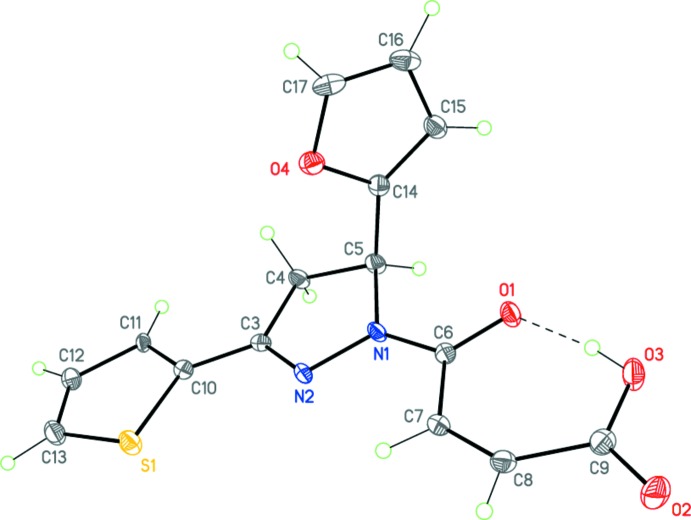
The mol­ecular structure of (II). Displacement ellipsoids are shown at the 50% probability level. H atoms are presented as small spheres of arbitrary radius. The dashed line indicates the intra­molecular O—H⋯O hydrogen bond.

**Table 1:** Hydrogen-bond geometry (Å, °) for (I)

*D*—H⋯*A*	*D*—H	H⋯*A*	*D*⋯*A*	*D*—H⋯*A*
O3—H3⋯O1	1.02 (3)	1.50 (3)	2.513 (2)	171 (2)
C19—H19⋯O2^i^	0.95	2.40	3.266 (3)	152

Symmetry code: (i) 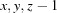.

**Table 2:** Hydrogen-bond geometry (Å, °) for (II)

*D*—H⋯*A*	*D*—H	H⋯*A*	*D*⋯*A*	*D*—H⋯*A*
O3—H3⋯O1	0.88 (3)	1.64 (3)	2.5146 (19)	171 (2)
C4—H4*B*⋯S1^i^	0.99	2.85	3.820 (2)	165
C17—H17⋯O1^ii^	0.95	2.51	3.426 (2)	161

Symmetry codes: (i) 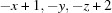; (ii) 
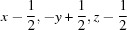
.

**Table 3:** Experimental details

	(I)	(II)
Crystal data		
Chemical formula	C_17_H_14_N_2_O_4_	C_15_H_12_N_2_O_4_S
*M*_r_	310.30	316.33
Crystal system, space group	Triclinic, *P* 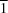	Monoclinic, *P*2_1_/*n*
Temperature (K)	100	100
*a*, *b*, *c* (Å)	7.2940 (15), 10.738 (2), 10.845 (2)	9.6702 (19), 13.150 (3), 11.240 (2)
α, β, γ (°)	114.10 (3), 102.46 (3), 97.57 (3)	90, 98.29 (3), 90
*V* (Å^3^)	733.8 (3)	1414.4 (5)
*Z*	2	4
Radiation type	Synchrotron, λ = 0.96990 Å	Synchrotron, λ = 0.96990 Å
μ (mm^−1^)	0.22	0.58
Crystal size (mm)	0.20 × 0.07 × 0.07	0.40 × 0.30 × 0.20

Data collection		
Diffractometer	MAR CCD	MAR CCD
Absorption correction	Multi-scan (*SCALA*; Evans, 2006 ▸)	Multi-scan (*SCALA*; Evans, 2006 ▸)
*T*_min_, *T*_max_	0.950, 0.980	0.789, 0.876
No. of measured, independent and observed [*I* > 2σ(*I*)] reflections	10451, 2947, 2189	17279, 2961, 2685
*R*_int_	0.103	0.087
(sin θ/λ)_max_ (Å^−1^)	0.641	0.642

Refinement		
*R*[*F* ^2^ > 2σ(*F* ^2^)], *wR*(*F* ^2^), *S*	0.075, 0.199, 1.04	0.045, 0.119, 1.05
No. of reflections	2947	2961
No. of parameters	212	203
H-atom treatment	H atoms treated by a mixture of independent and constrained refinement	H atoms treated by a mixture of independent and constrained refinement
Δρ_max_, Δρ_min_ (e Å^−3^)	0.41, −0.39	0.43, −0.52

Computer programs: *Automar* (MarXperts, 2015 ▸), *i* *MOSFLM* (Battye *et al.*, 2011 ▸), *SHELXS97* and *SHELXTL* (Sheldrick, 2008 ▸) and *SHELXL2014* (Sheldrick, 2015 ▸).

## supplementary crystallographic information

### (I) (5RS)-(Z)-4-[5-(Furan-2-yl)-3-phenyl-4,5-dihydro-1H-pyrazol-1-yl]-4-oxobut-2-enoic acid Crystal data

**Table:**

C_17_H_14_N_2_O_4_	*Z* = 2
*M**_r_* = 310.30	*F*(000) = 324
Triclinic, *P*1	*D*_x_ = 1.404 Mg m^−^^3^
*a* = 7.2940 (15) Å	Synchrotron radiation, λ = 0.96990 Å
*b* = 10.738 (2) Å	Cell parameters from 500 reflections
*c* = 10.845 (2) Å	θ = 4.0–36.0°
α = 114.10 (3)°	µ = 0.22 mm^−^^1^
β = 102.46 (3)°	*T* = 100 K
γ = 97.57 (3)°	Needle, colourless
*V* = 733.8 (3) Å^3^	0.20 × 0.07 × 0.07 mm

### (I) (5RS)-(Z)-4-[5-(Furan-2-yl)-3-phenyl-4,5-dihydro-1H-pyrazol-1-yl]-4-oxobut-2-enoic acid Data collection

**Table:**

MAR CCD diffractometer	2189 reflections with *I* > 2σ(*I*)
/f scan	*R*_int_ = 0.103
Absorption correction: multi-scan (SCALA; Evans, 2006)	θ_max_ = 38.4°, θ_min_ = 4.0°
*T*_min_ = 0.950, *T*_max_ = 0.980	*h* = −9→9
10451 measured reflections	*k* = −13→12
2947 independent reflections	*l* = −13→13

### (I) (5RS)-(Z)-4-[5-(Furan-2-yl)-3-phenyl-4,5-dihydro-1H-pyrazol-1-yl]-4-oxobut-2-enoic acid Refinement

**Table:**

Refinement on *F*^2^	Secondary atom site location: difference Fourier map
Least-squares matrix: full	Hydrogen site location: inferred from neighbouring sites
*R*[*F*^2^ > 2σ(*F*^2^)] = 0.075	H atoms treated by a mixture of independent and constrained refinement
*wR*(*F*^2^) = 0.199	*w* = 1/[σ^2^(*F*_o_^2^) + (0.088*P*)^2^] where *P* = (*F*_o_^2^ + 2*F*_c_^2^)/3
*S* = 1.04	(Δ/σ)_max_ < 0.001
2947 reflections	Δρ_max_ = 0.41 e Å^−^^3^
212 parameters	Δρ_min_ = −0.39 e Å^−^^3^
0 restraints	Extinction correction: SHELXL2014 (Sheldrick, 2015), Fc^*^=kFc[1+0.001xFc^2^λ^3^/sin(2θ)]^-1/4^
Primary atom site location: difference Fourier map	Extinction coefficient: 0.112 (13)

### (I) (5RS)-(Z)-4-[5-(Furan-2-yl)-3-phenyl-4,5-dihydro-1H-pyrazol-1-yl]-4-oxobut-2-enoic acid Special details

**Table:**

Geometry. All esds (except the esd in the dihedral angle between two l.s. planes) are estimated using the full covariance matrix. The cell esds are taken into account individually in the estimation of esds in distances, angles and torsion angles; correlations between esds in cell parameters are only used when they are defined by crystal symmetry. An approximate (isotropic) treatment of cell esds is used for estimating esds involving l.s. planes.

### (I) (5RS)-(Z)-4-[5-(Furan-2-yl)-3-phenyl-4,5-dihydro-1H-pyrazol-1-yl]-4-oxobut-2-enoic acid Fractional atomic coordinates and isotropic or equivalent isotropic displacement parameters (Å^2^)

**Table:**

	*x*	*y*	*z*	*U*_iso_*/*U*_eq_	
O1	0.53546 (18)	0.85698 (13)	0.87765 (13)	0.0294 (4)	
O2	0.8189 (2)	0.71553 (16)	1.17671 (15)	0.0402 (5)	
O3	0.7068 (2)	0.86788 (15)	1.11054 (15)	0.0351 (5)	
H3	0.644 (3)	0.874 (2)	1.020 (3)	0.053*	
O4	0.55732 (19)	0.79397 (15)	0.46578 (14)	0.0329 (5)	
N1	0.3716 (2)	0.69540 (16)	0.65583 (16)	0.0249 (5)	
N2	0.2966 (2)	0.55549 (16)	0.54965 (17)	0.0256 (5)	
C3	0.1865 (2)	0.5590 (2)	0.4406 (2)	0.0242 (5)	
C4	0.1695 (3)	0.7049 (2)	0.4619 (2)	0.0275 (5)	
H4A	0.0397	0.7182	0.4690	0.033*	
H4B	0.1943	0.7229	0.3837	0.033*	
C5	0.3284 (3)	0.8010 (2)	0.6030 (2)	0.0269 (5)	
H5	0.2761	0.8733	0.6688	0.032*	
C6	0.4790 (2)	0.7294 (2)	0.7886 (2)	0.0259 (5)	
C7	0.5227 (3)	0.6115 (2)	0.8181 (2)	0.0266 (5)	
H7	0.4700	0.5198	0.7415	0.032*	
C8	0.6277 (3)	0.6188 (2)	0.9400 (2)	0.0286 (5)	
H8	0.6429	0.5301	0.9337	0.034*	
C9	0.7259 (3)	0.7393 (2)	1.0843 (2)	0.0293 (6)	
C10	0.0858 (2)	0.4281 (2)	0.30984 (19)	0.0254 (5)	
C11	−0.0296 (3)	0.4332 (2)	0.1915 (2)	0.0278 (5)	
H11	−0.0446	0.5212	0.1955	0.033*	
C12	−0.1226 (3)	0.3089 (2)	0.0676 (2)	0.0304 (6)	
H12	−0.1982	0.3128	−0.0133	0.036*	
C13	−0.1049 (3)	0.1801 (2)	0.0623 (2)	0.0319 (6)	
H13	−0.1711	0.0958	−0.0215	0.038*	
C14	0.0096 (3)	0.1735 (2)	0.1793 (2)	0.0307 (6)	
H14	0.0216	0.0849	0.1751	0.037*	
C15	0.1064 (3)	0.2973 (2)	0.3027 (2)	0.0268 (5)	
H15	0.1864	0.2931	0.3819	0.032*	
C16	0.5068 (3)	0.8691 (2)	0.5855 (2)	0.0268 (5)	
C17	0.6447 (3)	0.9908 (2)	0.6709 (2)	0.0311 (6)	
H17	0.6450	1.0607	0.7600	0.037*	
C18	0.7893 (3)	0.9938 (2)	0.6013 (2)	0.0374 (6)	
H18	0.9041	1.0661	0.6349	0.045*	
C19	0.7316 (3)	0.8742 (2)	0.4787 (2)	0.0374 (6)	
H19	0.8007	0.8486	0.4109	0.045*	

### (I) (5RS)-(Z)-4-[5-(Furan-2-yl)-3-phenyl-4,5-dihydro-1H-pyrazol-1-yl]-4-oxobut-2-enoic acid Atomic displacement parameters (Å^2^)

**Table:**

	*U*^11^	*U*^22^	*U*^33^	*U*^12^	*U*^13^	*U*^23^
O1	0.0309 (7)	0.0236 (8)	0.0258 (8)	0.0015 (6)	0.0020 (6)	0.0084 (6)
O2	0.0480 (9)	0.0376 (9)	0.0283 (9)	0.0095 (7)	−0.0028 (7)	0.0158 (7)
O3	0.0433 (9)	0.0276 (9)	0.0251 (8)	0.0041 (6)	−0.0018 (7)	0.0105 (7)
O4	0.0305 (8)	0.0355 (9)	0.0305 (9)	0.0058 (6)	0.0069 (6)	0.0146 (7)
N1	0.0260 (8)	0.0210 (9)	0.0214 (9)	0.0011 (7)	0.0002 (7)	0.0085 (7)
N2	0.0249 (8)	0.0232 (9)	0.0228 (9)	0.0021 (7)	0.0034 (7)	0.0078 (7)
C3	0.0211 (9)	0.0262 (11)	0.0237 (11)	0.0041 (8)	0.0065 (8)	0.0105 (9)
C4	0.0229 (9)	0.0286 (11)	0.0248 (11)	0.0021 (8)	0.0008 (8)	0.0106 (9)
C5	0.0260 (9)	0.0263 (11)	0.0246 (10)	0.0043 (8)	0.0004 (8)	0.0122 (9)
C6	0.0216 (9)	0.0295 (11)	0.0244 (11)	0.0027 (8)	0.0030 (8)	0.0132 (9)
C7	0.0274 (9)	0.0248 (11)	0.0240 (10)	0.0020 (8)	0.0053 (8)	0.0099 (9)
C8	0.0280 (10)	0.0287 (11)	0.0276 (11)	0.0035 (8)	0.0039 (8)	0.0145 (9)
C9	0.0302 (10)	0.0293 (12)	0.0266 (11)	0.0043 (8)	0.0039 (8)	0.0140 (9)
C10	0.0218 (9)	0.0295 (12)	0.0234 (11)	0.0032 (8)	0.0051 (8)	0.0122 (9)
C11	0.0255 (9)	0.0293 (11)	0.0259 (11)	0.0041 (8)	0.0037 (8)	0.0128 (9)
C12	0.0283 (10)	0.0326 (12)	0.0237 (11)	0.0043 (9)	0.0019 (8)	0.0105 (9)
C13	0.0288 (10)	0.0315 (12)	0.0254 (11)	0.0025 (8)	0.0022 (8)	0.0079 (9)
C14	0.0293 (10)	0.0275 (11)	0.0325 (12)	0.0052 (8)	0.0064 (9)	0.0127 (9)
C15	0.0243 (9)	0.0275 (11)	0.0260 (11)	0.0040 (8)	0.0043 (8)	0.0119 (9)
C16	0.0279 (10)	0.0242 (11)	0.0246 (10)	0.0040 (8)	0.0005 (8)	0.0118 (9)
C17	0.0300 (10)	0.0250 (11)	0.0283 (11)	−0.0009 (8)	−0.0015 (8)	0.0100 (9)
C18	0.0252 (10)	0.0400 (13)	0.0512 (14)	0.0009 (9)	0.0019 (9)	0.0314 (12)
C19	0.0283 (10)	0.0525 (15)	0.0437 (14)	0.0105 (10)	0.0103 (9)	0.0334 (12)

### (I) (5RS)-(Z)-4-[5-(Furan-2-yl)-3-phenyl-4,5-dihydro-1H-pyrazol-1-yl]-4-oxobut-2-enoic acid Geometric parameters (Å, º)

**Table:**

O1—C6	1.256 (2)	C8—C9	1.501 (3)
O2—C9	1.222 (2)	C8—H8	0.9500
O3—C9	1.324 (3)	C10—C11	1.400 (3)
O3—H3	1.02 (3)	C10—C15	1.405 (3)
O4—C16	1.379 (3)	C11—C12	1.396 (3)
O4—C19	1.384 (2)	C11—H11	0.9500
N1—C6	1.352 (2)	C12—C13	1.386 (3)
N1—N2	1.408 (2)	C12—H12	0.9500
N1—C5	1.504 (2)	C13—C14	1.395 (3)
N2—C3	1.295 (2)	C13—H13	0.9500
C3—C10	1.475 (3)	C14—C15	1.396 (3)
C3—C4	1.515 (3)	C14—H14	0.9500
C4—C5	1.542 (3)	C15—H15	0.9500
C4—H4A	0.9900	C16—C17	1.357 (3)
C4—H4B	0.9900	C17—C18	1.428 (3)
C5—C16	1.494 (3)	C17—H17	0.9500
C5—H5	1.0000	C18—C19	1.348 (3)
C6—C7	1.483 (3)	C18—H18	0.9500
C7—C8	1.343 (3)	C19—H19	0.9500
C7—H7	0.9500		
			
C9—O3—H3	111.6 (14)	O3—C9—C8	120.10 (18)
C16—O4—C19	106.22 (16)	C11—C10—C15	119.44 (17)
C6—N1—N2	122.90 (16)	C11—C10—C3	120.42 (19)
C6—N1—C5	124.32 (15)	C15—C10—C3	120.14 (17)
N2—N1—C5	112.71 (14)	C12—C11—C10	120.0 (2)
C3—N2—N1	107.28 (16)	C12—C11—H11	120.0
N2—C3—C10	120.85 (18)	C10—C11—H11	120.0
N2—C3—C4	114.48 (16)	C13—C12—C11	120.21 (19)
C10—C3—C4	124.66 (16)	C13—C12—H12	119.9
C3—C4—C5	102.65 (15)	C11—C12—H12	119.9
C3—C4—H4A	111.2	C12—C13—C14	120.36 (18)
C5—C4—H4A	111.2	C12—C13—H13	119.8
C3—C4—H4B	111.2	C14—C13—H13	119.8
C5—C4—H4B	111.2	C13—C14—C15	119.85 (19)
H4A—C4—H4B	109.1	C13—C14—H14	120.1
C16—C5—N1	110.07 (15)	C15—C14—H14	120.1
C16—C5—C4	113.66 (17)	C14—C15—C10	120.09 (18)
N1—C5—C4	100.45 (14)	C14—C15—H15	120.0
C16—C5—H5	110.8	C10—C15—H15	120.0
N1—C5—H5	110.8	C17—C16—O4	109.89 (18)
C4—C5—H5	110.8	C17—C16—C5	132.7 (2)
O1—C6—N1	118.52 (17)	O4—C16—C5	117.25 (16)
O1—C6—C7	124.58 (16)	C16—C17—C18	106.85 (19)
N1—C6—C7	116.90 (17)	C16—C17—H17	126.6
C8—C7—C6	127.84 (18)	C18—C17—H17	126.6
C8—C7—H7	116.1	C19—C18—C17	106.76 (18)
C6—C7—H7	116.1	C19—C18—H18	126.6
C7—C8—C9	132.8 (2)	C17—C18—H18	126.6
C7—C8—H8	113.6	C18—C19—O4	110.3 (2)
C9—C8—H8	113.6	C18—C19—H19	124.9
O2—C9—O3	121.15 (18)	O4—C19—H19	124.9
O2—C9—C8	118.72 (19)		
			
C6—N1—N2—C3	173.99 (17)	C4—C3—C10—C11	2.7 (3)
C5—N1—N2—C3	−9.2 (2)	N2—C3—C10—C15	0.8 (3)
N1—N2—C3—C10	179.71 (16)	C4—C3—C10—C15	−177.85 (17)
N1—N2—C3—C4	−1.5 (2)	C15—C10—C11—C12	−0.2 (3)
N2—C3—C4—C5	10.8 (2)	C3—C10—C11—C12	179.26 (18)
C10—C3—C4—C5	−170.49 (19)	C10—C11—C12—C13	1.5 (3)
C6—N1—C5—C16	71.7 (2)	C11—C12—C13—C14	−1.5 (3)
N2—N1—C5—C16	−105.08 (17)	C12—C13—C14—C15	0.2 (3)
C6—N1—C5—C4	−168.17 (19)	C13—C14—C15—C10	1.2 (3)
N2—N1—C5—C4	15.0 (2)	C11—C10—C15—C14	−1.2 (3)
C3—C4—C5—C16	103.36 (18)	C3—C10—C15—C14	179.38 (18)
C3—C4—C5—N1	−14.14 (18)	C19—O4—C16—C17	−0.7 (2)
N2—N1—C6—O1	−178.08 (16)	C19—O4—C16—C5	−176.11 (16)
C5—N1—C6—O1	5.4 (3)	N1—C5—C16—C17	−95.2 (3)
N2—N1—C6—C7	2.3 (3)	C4—C5—C16—C17	153.0 (2)
C5—N1—C6—C7	−174.19 (17)	N1—C5—C16—O4	78.9 (2)
O1—C6—C7—C8	−0.6 (3)	C4—C5—C16—O4	−32.8 (2)
N1—C6—C7—C8	179.0 (2)	O4—C16—C17—C18	0.6 (2)
C6—C7—C8—C9	3.2 (4)	C5—C16—C17—C18	175.1 (2)
C7—C8—C9—O2	−177.6 (2)	C16—C17—C18—C19	−0.3 (2)
C7—C8—C9—O3	4.1 (4)	C17—C18—C19—O4	−0.2 (2)
N2—C3—C10—C11	−178.62 (16)	C16—O4—C19—C18	0.5 (2)

### (I) (5RS)-(Z)-4-[5-(Furan-2-yl)-3-phenyl-4,5-dihydro-1H-pyrazol-1-yl]-4-oxobut-2-enoic acid Hydrogen-bond geometry (Å, º)

**Table:**

*D*—H···*A*	*D*—H	H···*A*	*D*···*A*	*D*—H···*A*
O3—H3···O1	1.02 (3)	1.50 (3)	2.513 (2)	171 (2)
C19—H19···O2^i^	0.95	2.40	3.266 (3)	152

Symmetry code: (i) *x*, *y*, *z*−1.

### (II) (5RS)-(Z)-4-[5-(Furan-2-yl)-3-(thiophen-2-yl)-4,5-dihydro-1H-pyrazol-1-yl]-4-oxobut-2-enoic acid Crystal data

**Table:**

C_15_H_12_N_2_O_4_S	*F*(000) = 656
*M**_r_* = 316.33	*D*_x_ = 1.485 Mg m^−^^3^
Monoclinic, *P*2_1_/*n*	Synchrotron radiation, λ = 0.96990 Å
*a* = 9.6702 (19) Å	Cell parameters from 600 reflections
*b* = 13.150 (3) Å	θ = 3.6–36.0°
*c* = 11.240 (2) Å	µ = 0.58 mm^−^^1^
β = 98.29 (3)°	*T* = 100 K
*V* = 1414.4 (5) Å^3^	Prism, colourless
*Z* = 4	0.40 × 0.30 × 0.20 mm

### (II) (5RS)-(Z)-4-[5-(Furan-2-yl)-3-(thiophen-2-yl)-4,5-dihydro-1H-pyrazol-1-yl]-4-oxobut-2-enoic acid Data collection

**Table:**

MAR CCD diffractometer	2685 reflections with *I* > 2σ(*I*)
/f scan	*R*_int_ = 0.087
Absorption correction: multi-scan (SCALA; Evans, 2006)	θ_max_ = 38.5°, θ_min_ = 3.6°
*T*_min_ = 0.789, *T*_max_ = 0.876	*h* = −12→12
17279 measured reflections	*k* = −16→14
2961 independent reflections	*l* = −13→14

### (II) (5RS)-(Z)-4-[5-(Furan-2-yl)-3-(thiophen-2-yl)-4,5-dihydro-1H-pyrazol-1-yl]-4-oxobut-2-enoic acid Refinement

**Table:**

Refinement on *F*^2^	Secondary atom site location: difference Fourier map
Least-squares matrix: full	Hydrogen site location: inferred from neighbouring sites
*R*[*F*^2^ > 2σ(*F*^2^)] = 0.045	H atoms treated by a mixture of independent and constrained refinement
*wR*(*F*^2^) = 0.119	*w* = 1/[σ^2^(*F*_o_^2^) + (0.0506*P*)^2^ + 1.1417*P*] where *P* = (*F*_o_^2^ + 2*F*_c_^2^)/3
*S* = 1.05	(Δ/σ)_max_ < 0.001
2961 reflections	Δρ_max_ = 0.43 e Å^−^^3^
203 parameters	Δρ_min_ = −0.52 e Å^−^^3^
0 restraints	Extinction correction: SHELXL2014 (Sheldrick, 2015), Fc^*^=kFc[1+0.001xFc^2^λ^3^/sin(2θ)]^-1/4^
Primary atom site location: difference Fourier map	Extinction coefficient: 0.029 (2)

### (II) (5RS)-(Z)-4-[5-(Furan-2-yl)-3-(thiophen-2-yl)-4,5-dihydro-1H-pyrazol-1-yl]-4-oxobut-2-enoic acid Special details

**Table:**

Geometry. All esds (except the esd in the dihedral angle between two l.s. planes) are estimated using the full covariance matrix. The cell esds are taken into account individually in the estimation of esds in distances, angles and torsion angles; correlations between esds in cell parameters are only used when they are defined by crystal symmetry. An approximate (isotropic) treatment of cell esds is used for estimating esds involving l.s. planes.

### (II) (5RS)-(Z)-4-[5-(Furan-2-yl)-3-(thiophen-2-yl)-4,5-dihydro-1H-pyrazol-1-yl]-4-oxobut-2-enoic acid Fractional atomic coordinates and isotropic or equivalent isotropic displacement parameters (Å^2^)

**Table:**

	*x*	*y*	*z*	*U*_iso_*/*U*_eq_	
S1	0.18979 (4)	0.02971 (3)	0.94264 (4)	0.01637 (17)	
O1	0.62416 (12)	0.37278 (9)	0.76273 (11)	0.0158 (3)	
O2	0.46922 (14)	0.66885 (9)	0.86341 (12)	0.0226 (3)	
O3	0.60922 (13)	0.56265 (10)	0.78427 (12)	0.0206 (3)	
H3	0.621 (3)	0.497 (2)	0.772 (2)	0.031*	
O4	0.44618 (12)	0.14434 (10)	0.54975 (11)	0.0165 (3)	
N1	0.50566 (14)	0.23234 (10)	0.79925 (12)	0.0118 (3)	
N2	0.40101 (14)	0.17978 (11)	0.84886 (12)	0.0120 (3)	
C3	0.42845 (16)	0.08325 (12)	0.84155 (14)	0.0114 (3)	
C4	0.55685 (17)	0.05821 (13)	0.78427 (15)	0.0133 (3)	
H4A	0.5342	0.0104	0.7162	0.016*	
H4B	0.6316	0.0285	0.8436	0.016*	
C5	0.59983 (16)	0.16366 (12)	0.74062 (15)	0.0124 (3)	
H5	0.6999	0.1781	0.7723	0.015*	
C6	0.52109 (17)	0.33450 (12)	0.80326 (15)	0.0117 (3)	
C7	0.41319 (17)	0.39413 (13)	0.85407 (15)	0.0134 (3)	
H7	0.3409	0.3558	0.8819	0.016*	
C8	0.40489 (18)	0.49611 (13)	0.86571 (15)	0.0147 (4)	
H8	0.3246	0.5181	0.8984	0.018*	
C9	0.49805 (18)	0.58175 (13)	0.83701 (15)	0.0158 (4)	
C10	0.34310 (17)	0.00445 (13)	0.88510 (14)	0.0123 (3)	
C11	0.37250 (16)	−0.10024 (12)	0.88526 (14)	0.0107 (3)	
H11	0.4522	−0.1292	0.8579	0.013*	
C12	0.26597 (18)	−0.15765 (13)	0.93240 (16)	0.0154 (4)	
H12	0.2672	−0.2296	0.9397	0.019*	
C13	0.16253 (18)	−0.09732 (14)	0.96569 (16)	0.0178 (4)	
H13	0.0841	−0.1231	0.9981	0.021*	
C14	0.57458 (17)	0.17614 (12)	0.60747 (15)	0.0125 (3)	
C15	0.65215 (18)	0.21555 (13)	0.52703 (16)	0.0162 (4)	
H15	0.7443	0.2419	0.5440	0.019*	
C16	0.56731 (19)	0.20951 (14)	0.41102 (16)	0.0189 (4)	
H16	0.5922	0.2313	0.3364	0.023*	
C17	0.44516 (19)	0.16660 (14)	0.42938 (16)	0.0188 (4)	
H17	0.3690	0.1535	0.3680	0.023*	

### (II) (5RS)-(Z)-4-[5-(Furan-2-yl)-3-(thiophen-2-yl)-4,5-dihydro-1H-pyrazol-1-yl]-4-oxobut-2-enoic acid Atomic displacement parameters (Å^2^)

**Table:**

	*U*^11^	*U*^22^	*U*^33^	*U*^12^	*U*^13^	*U*^23^
S1	0.0148 (3)	0.0149 (3)	0.0206 (3)	0.00060 (15)	0.00654 (18)	0.00097 (15)
O1	0.0136 (6)	0.0140 (6)	0.0213 (7)	−0.0020 (4)	0.0077 (5)	0.0004 (5)
O2	0.0310 (8)	0.0120 (6)	0.0251 (7)	0.0012 (5)	0.0054 (6)	−0.0003 (5)
O3	0.0192 (6)	0.0131 (6)	0.0311 (8)	−0.0036 (5)	0.0087 (5)	−0.0009 (5)
O4	0.0129 (6)	0.0211 (6)	0.0155 (6)	0.0007 (5)	0.0024 (5)	−0.0019 (5)
N1	0.0115 (7)	0.0111 (7)	0.0144 (7)	−0.0004 (5)	0.0072 (5)	0.0005 (5)
N2	0.0119 (7)	0.0123 (7)	0.0128 (7)	−0.0027 (5)	0.0052 (5)	0.0007 (5)
C3	0.0124 (7)	0.0129 (8)	0.0091 (8)	0.0004 (6)	0.0023 (6)	−0.0005 (6)
C4	0.0136 (8)	0.0132 (8)	0.0143 (8)	0.0014 (6)	0.0062 (6)	0.0007 (6)
C5	0.0105 (7)	0.0118 (8)	0.0160 (8)	0.0022 (6)	0.0061 (6)	−0.0007 (6)
C6	0.0115 (8)	0.0120 (8)	0.0115 (8)	−0.0002 (6)	0.0014 (6)	0.0004 (6)
C7	0.0131 (8)	0.0145 (8)	0.0136 (8)	−0.0008 (6)	0.0049 (6)	−0.0008 (6)
C8	0.0154 (8)	0.0157 (8)	0.0137 (8)	0.0009 (7)	0.0042 (6)	−0.0009 (7)
C9	0.0194 (8)	0.0141 (8)	0.0134 (8)	0.0002 (7)	0.0007 (6)	0.0009 (6)
C10	0.0131 (8)	0.0123 (8)	0.0118 (8)	−0.0009 (6)	0.0025 (6)	−0.0004 (6)
C11	0.0118 (7)	0.0109 (7)	0.0093 (7)	−0.0043 (6)	0.0006 (6)	0.0017 (6)
C12	0.0180 (8)	0.0130 (8)	0.0158 (8)	−0.0030 (6)	0.0041 (6)	0.0012 (6)
C13	0.0159 (8)	0.0183 (9)	0.0203 (9)	−0.0028 (7)	0.0062 (7)	0.0011 (7)
C14	0.0110 (7)	0.0119 (8)	0.0152 (8)	0.0016 (6)	0.0035 (6)	−0.0011 (6)
C15	0.0178 (8)	0.0140 (8)	0.0181 (9)	0.0003 (6)	0.0073 (7)	0.0004 (6)
C16	0.0278 (9)	0.0163 (8)	0.0138 (9)	0.0059 (7)	0.0072 (7)	0.0007 (7)
C17	0.0228 (9)	0.0204 (9)	0.0125 (9)	0.0072 (7)	0.0004 (7)	−0.0033 (7)

### (II) (5RS)-(Z)-4-[5-(Furan-2-yl)-3-(thiophen-2-yl)-4,5-dihydro-1H-pyrazol-1-yl]-4-oxobut-2-enoic acid Geometric parameters (Å, º)

**Table:**

S1—C13	1.7166 (19)	C5—H5	1.0000
S1—C10	1.7330 (17)	C6—C7	1.484 (2)
O1—C6	1.2592 (19)	C7—C8	1.351 (2)
O2—C9	1.225 (2)	C7—H7	0.9500
O3—C9	1.324 (2)	C8—C9	1.506 (2)
O3—H3	0.88 (3)	C8—H8	0.9500
O4—C14	1.380 (2)	C10—C11	1.406 (2)
O4—C17	1.383 (2)	C11—C12	1.439 (2)
N1—C6	1.352 (2)	C11—H11	0.9500
N1—N2	1.4056 (17)	C12—C13	1.370 (2)
N1—C5	1.5007 (19)	C12—H12	0.9500
N2—C3	1.302 (2)	C13—H13	0.9500
C3—C10	1.453 (2)	C14—C15	1.358 (2)
C3—C4	1.515 (2)	C15—C16	1.439 (3)
C4—C5	1.548 (2)	C15—H15	0.9500
C4—H4A	0.9900	C16—C17	1.351 (3)
C4—H4B	0.9900	C16—H16	0.9500
C5—C14	1.490 (2)	C17—H17	0.9500
			
C13—S1—C10	91.61 (8)	C7—C8—H8	113.7
C9—O3—H3	113.0 (16)	C9—C8—H8	113.7
C14—O4—C17	105.94 (13)	O2—C9—O3	120.88 (16)
C6—N1—N2	123.90 (13)	O2—C9—C8	118.89 (16)
C6—N1—C5	122.81 (13)	O3—C9—C8	120.23 (15)
N2—N1—C5	113.29 (12)	C11—C10—C3	125.05 (15)
C3—N2—N1	106.83 (12)	C11—C10—S1	111.78 (12)
N2—C3—C10	122.91 (14)	C3—C10—S1	123.17 (13)
N2—C3—C4	115.19 (14)	C10—C11—C12	111.03 (14)
C10—C3—C4	121.90 (14)	C10—C11—H11	124.5
C3—C4—C5	102.36 (13)	C12—C11—H11	124.5
C3—C4—H4A	111.3	C13—C12—C11	112.74 (15)
C5—C4—H4A	111.3	C13—C12—H12	123.6
C3—C4—H4B	111.3	C11—C12—H12	123.6
C5—C4—H4B	111.3	C12—C13—S1	112.84 (13)
H4A—C4—H4B	109.2	C12—C13—H13	123.6
C14—C5—N1	110.66 (13)	S1—C13—H13	123.6
C14—C5—C4	113.85 (14)	C15—C14—O4	110.40 (15)
N1—C5—C4	101.09 (12)	C15—C14—C5	133.19 (16)
C14—C5—H5	110.3	O4—C14—C5	116.40 (14)
N1—C5—H5	110.3	C14—C15—C16	106.54 (15)
C4—C5—H5	110.3	C14—C15—H15	126.7
O1—C6—N1	118.33 (14)	C16—C15—H15	126.7
O1—C6—C7	124.42 (15)	C17—C16—C15	106.33 (16)
N1—C6—C7	117.24 (14)	C17—C16—H16	126.8
C8—C7—C6	128.08 (15)	C15—C16—H16	126.8
C8—C7—H7	116.0	C16—C17—O4	110.78 (16)
C6—C7—H7	116.0	C16—C17—H17	124.6
C7—C8—C9	132.52 (16)	O4—C17—H17	124.6
			
C6—N1—N2—C3	−172.26 (15)	C4—C3—C10—C11	3.5 (2)
C5—N1—N2—C3	7.76 (17)	N2—C3—C10—S1	4.2 (2)
N1—N2—C3—C10	179.46 (14)	C4—C3—C10—S1	−175.93 (12)
N1—N2—C3—C4	−0.40 (18)	C13—S1—C10—C11	−0.62 (13)
N2—C3—C4—C5	−6.46 (19)	C13—S1—C10—C3	178.89 (14)
C10—C3—C4—C5	173.68 (14)	C3—C10—C11—C12	−178.98 (15)
C6—N1—C5—C14	−70.26 (19)	S1—C10—C11—C12	0.51 (17)
N2—N1—C5—C14	109.72 (15)	C10—C11—C12—C13	−0.1 (2)
C6—N1—C5—C4	168.79 (14)	C11—C12—C13—S1	−0.4 (2)
N2—N1—C5—C4	−11.23 (17)	C10—S1—C13—C12	0.57 (15)
C3—C4—C5—C14	−108.98 (15)	C17—O4—C14—C15	−0.77 (18)
C3—C4—C5—N1	9.68 (16)	C17—O4—C14—C5	177.90 (13)
N2—N1—C6—O1	175.44 (14)	N1—C5—C14—C15	111.5 (2)
C5—N1—C6—O1	−4.6 (2)	C4—C5—C14—C15	−135.49 (19)
N2—N1—C6—C7	−5.4 (2)	N1—C5—C14—O4	−66.83 (17)
C5—N1—C6—C7	174.58 (14)	C4—C5—C14—O4	46.22 (19)
O1—C6—C7—C8	0.2 (3)	O4—C14—C15—C16	0.66 (18)
N1—C6—C7—C8	−178.88 (17)	C5—C14—C15—C16	−177.71 (17)
C6—C7—C8—C9	−2.1 (3)	C14—C15—C16—C17	−0.28 (19)
C7—C8—C9—O2	−175.82 (18)	C15—C16—C17—O4	−0.2 (2)
C7—C8—C9—O3	4.1 (3)	C14—O4—C17—C16	0.59 (19)
N2—C3—C10—C11	−176.34 (15)		

### (II) (5RS)-(Z)-4-[5-(Furan-2-yl)-3-(thiophen-2-yl)-4,5-dihydro-1H-pyrazol-1-yl]-4-oxobut-2-enoic acid Hydrogen-bond geometry (Å, º)

**Table:**

*D*—H···*A*	*D*—H	H···*A*	*D*···*A*	*D*—H···*A*
O3—H3···O1	0.88 (3)	1.64 (3)	2.5146 (19)	171 (2)
C4—H4*B*···S1^i^	0.99	2.85	3.820 (2)	165
C17—H17···O1^ii^	0.95	2.51	3.426 (2)	161

Symmetry codes: (i) −*x*+1, −*y*, −*z*+2; (ii) *x*−1/2, −*y*+1/2, *z*−1/2.

## References

[bb1] Ahmad, P., Woo, H., Jun, K.-Y., Kadi, A. A., Abdel-Aziz, H. A., Kwon, Y. & Rahman, A. F. M. M. (2016). *Bioorg. Med. Chem.* **24**, 1898–1908. 10.1016/j.bmc.2016.03.017 26988802

[bb2] Banday, A. H., Shameem, S. A. & Jeelani, S. (2014). *Steroids*, **92**, 13–19. 10.1016/j.steroids.2014.09.004 25278254

[bb3] Battye, T. G. G., Kontogiannis, L., Johnson, O., Powell, H. R. & Leslie, A. G. W. (2011). *Acta Cryst.* D**67**, 271–281. 10.1107/S0907444910048675 PMC3069742 21460445

[bb4] Bhoot, D., Khunt, R. C. & Parekh, H. H. (2012). *Med. Chem. Res.* **21**, 3233–3239.

[bb5] Cetin, A., Cansiz, A. & Digrak, M. (2003). *Heteroat. Chem.* **14**, 345–347.

[bb6] Deng, H., Yu, Z.-Y., Shi, G.-Y., Chen, M.-J., Tao, K. & Hou, T.-P. (2012). *Chem. Biol. Drug Des.* **79**, 279–289. 10.1111/j.1747-0285.2011.01308.x 22181692

[bb7] Evans, P. (2006). *Acta Cryst.* D**62**, 72–82. 10.1107/S0907444905036693 16369096

[bb8] Grandberg, I. I., Kost, A. N. & Sibiryakova, D. V. (1960). *Rus. J. Gen. Chem.* **30**, 2920–2925.

[bb9] Guo, H.-M. (2007). *Acta Cryst.* E**63**, o3165.

[bb10] Joshi, S. D., Dixit, S. R., Kirankumar, M. N., Aminabhavi, T. M., Raju, K. V. S. N., Narayan, R., Lherbet, C. & Yang, K. S. (2016). *Eur. J. Med. Chem.* **107**, 133–152. 10.1016/j.ejmech.2015.10.047 26580979

[bb11] Kriven’ko, A. P., Zapara, A. G., Ivannikov, A. V. & Klochkova, I. N. (2000). *Chem. Heterocycl. Compd.* **36**, 399–402.

[bb12] Suponitsky, K. Yu., Gusev, D. V., Kuleshova, L. N. & Antipin, M. Yu. (2002). *Crystallogr. Rep.* **47**, 667–671.

[bb13] Manna, K. & Agrawal, Y. K. (2010). *Eur. J. Med. Chem.* **45**, 3831–3839. 10.1016/j.ejmech.2010.05.035 20576327

[bb14] MarXperts. (2015). *Automar*. marXperts GmbH, Norderstedt, Germany.

[bb15] Özdemir, Z., Kandilci, H. B., Gümüşel, B., Çalış, Ü. & Bilgin, A. A. (2007). *Eur. J. Med. Chem.* **42**, 373–379. 10.1016/j.ejmech.2006.09.006 17069933

[bb16] Sheldrick, G. M. (2008). *Acta Cryst.* A**64**, 112–122. 10.1107/S0108767307043930 18156677

[bb17] Sheldrick, G. M. (2015). *Acta Cryst.* C**71**, 3–8.

[bb18] Shoman, M. E., Abdel-Aziz, M., Aly, O. M., Farag, H. H. & Morsy, M. A. (2009). *Eur. J. Med. Chem.* **44**, 3068–3076. 10.1016/j.ejmech.2008.07.008 18722034

[bb19] Vinutha, N., Madan Kumar, S., Vidyashree Jois, B. S., Balakrishna, K., Lokanath, N. K. & Revannasiddaiah, D. (2013). *Acta Cryst.* E**69**, o1528. 10.1107/S1600536813024690 PMC3790397 24098216

